# Toxicological Aspects and Determination of the Main Components of Ayahuasca: A Critical Review

**DOI:** 10.3390/medicines6040106

**Published:** 2019-10-18

**Authors:** Ana Y. Simão, Joana Gonçalves, Ana Paula Duarte, Mário Barroso, Ana Clara Cristóvão, Eugenia Gallardo

**Affiliations:** 1 Centro de Investigação em Ciências da Saúde, Universidade da Beira Interior (CICS-UBI), 6200-506 Covilhã, Portugal; anaaysa95@gmail.com (A.Y.S.); janitagoncalves@hotmail.com (J.G.); apduarte@fcsaude.ubi.pt (A.P.D.); aclaracristovao@gmail.com (A.C.C.); 2 Laboratório de Fármaco-Toxicologia, UBIMedical, Universidade da Beira Interior, 6200-284 Covilhã, Portugal; 3 Serviço de Química e Toxicologia Forenses, Instituto de Medicina Legal e Ciências Forenses - Delegação do Sul, 1169-201 Lisboa, Portugal; mario.j.barroso@inmlcf.mj.pt; 4 NEUROSOV, UBIMedical, Universidade da Beira Interior, 6200-284 Covilhã, Portugal

**Keywords:** ayahuasca, toxicity, analytical determination

## Abstract

Ayahuasca is a psychoactive beverage prepared traditionally from a mixture of the leaves and stems of *Psychotria viridis* and *Banisteriopsis caapi*, respectively, being originally consumed by indigenous Amazonian tribes for ritual and medicinal purposes. Over the years, its use has spread to other populations as a means to personal growth and spiritual connection. Also, the recreational use of its isolated compounds has become prominent. The main compounds of this tea-like preparation are N,N-dimethyltryptamine (DMT), β-Carbolines, and harmala alkaloids, such as harmine, tetrahydroharmine, and harmaline. The latter are monoamine-oxidase inhibitors and are responsible for DMT psychoactive and hallucinogenic effects on the central nervous system. Although consumers defend its use, its metabolic effects and those on the central nervous system are not fully understood yet. The majority of studies regarding the effects of this beverage and of its individual compounds are based on in vivo experiments, clinical trials, and even surveys. This paper will not only address the toxicological aspects of the ayahuasca compounds but also perform a comprehensive and critical review on the analytical methods available for their determination in biological and non-biological specimens, with special focus on instrumental developments and sample preparation approaches.

## 1. Introduction

Ayahuasca is an entheogenic beverage that has been consumed for centuries, originally by South American populations, more specifically, by Amazonian indigenous groups, in spiritual rituals and ceremonies in the hope of obtaining further knowledge and divine invigoration [1,2,3].

The term “*Ayahuasca*” has a Quechua origin; “aya” means “spirit”, and “waska” means “vine”, that is to say, “vine of the souls” [4]. Although sometimes known as ayahuasca, it can also be referred as *hoasca*, *caapi*, *daime*, *yagé*, *natema* and several other local names in Brazil, Bolivia, Equator, and Peru [5]. The word itself can be applied to either the beverage or the vine that is used to produce the beverage [6].

Over time, specific churches devoted to the consumption of ayahuasca were created in order to hold shamanic ceremonies to non-indigenous Amazonian populations. The most dominant are Santo Daime and União do Vegetal (UDV), among others. These religions are a fusion of Christianism, Spiritualism, and other religions with African-Brazilian bases. The diffusion of these religious through Northern America, Europe, and Asia as also led to an increase of ayahuasca consumption, and thus concern for public health, toxicity, and possible therapeutic potential have risen [1,7,8,9].

The beverage itself is a tea-like/decoction preparation that commonly consists of a brew mixture of *Banisteriopsis caapi* vine and *Psychotria viridis* leaves [10]. Nevertheless, there are analogues which can be used for the same purpose, for example, instead of *P. viridis*, *Psychotria carthagenensis, Brugmansia suaveolens, Nicotiana tabacum, Malouetia tamarquina, Tabernaemontana spp., Brunfelsia, spp., Datura suaveolens, Iochroma fuchsioides, Juanulloa spp*. can be used. [11]. In substitution of *B. caapi*, besides the natural analogue *Peganum harmala* (Syrian Rue), some synthetic compounds can be used, such as harmine freebase/HCl, tetrahydroharmine freebase/HCl, and moclobemide [3,12,13]. In addition, some of the analogues used in the blending of ayahuasca can contain different psychotropic substances, such as nicotine and caffeine [14].

**Figure 1:**
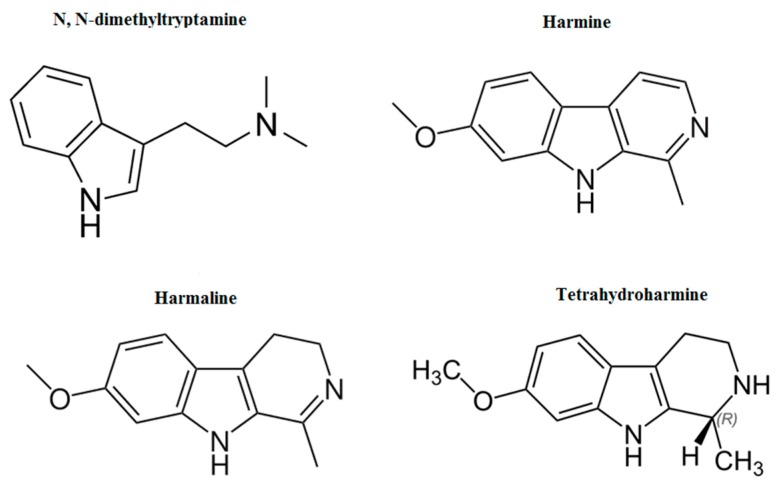
Chemical structures of N,N-dimethyltryptamine (DMT), tetrahydroharmine (THH), harmaline (HML), and harmine (HMN).

A scientific analysis of the *B. caapi*, a vine from the Malpighiaceaea family, showed that it is rich in β-carbolines alkaloids (B-CA), essentially harmine (HMN), tetrahydroharmine (THH), and harmaline (HML) [14,15]. Diversely, *P. viridis* belongs to the Rubiaceous family and contains N,N-dimethyltryptamine (DMT) [5,16]. The chemical structures of ayahuasca compounds are shown in Figure 1.

Depending on the origin and development of the plants used in ayahuasca beverage preparation, the chemical composition may differ, both in quantity and in quality. In addition, the preparation procedure of the beverage, done by religious or non-religious groups, can also impact on ayahuasca’s composition and ultimate effects [17]. Moreover, depending on the dose, the alkaloids present in ayahuasca beverage can also trigger an effect on their own [12].

As previously mentioned, ayahuasca consumption has been increasing and spreading all over the world, in some cases due to its reported health benefits [14,18]. In fact, some studies have described that, following a single dose of the drink, a rapid decrease in depressive symptoms is observed, which can last up to three weeks [19,20]. Other studies revealed that ayahuasca intake can significantly diminish anxiety and panic states [19,21]. In circumstances of drug and alcohol abuse, it has also been stated that the beverage can reduce the abuse of such substances [6,22,23], reduce attention disorders and lack of focus [24], as well as lower physical pain, insomnia, irritability, and obsessive symptoms [25]. Nonetheless, less beneficial and adverse effects have also been described when ayahuasca is consumed, for instance diarrhea, nausea, and vomiting [26] or increases in heart rate, blood pressure, and rectal temperature [27]. Particularly, DMT can also induce psychological disorders, such as emotional suffering, visual hallucinations, and changes of perception, cognition, and affection [26,28]. Moreover, ayahuasca consumption over a large period of time can also lead to episodes of psychosis [26].

Regarding the legal status of ayahuasca, there is some controversy on the matter due to the fact that neither the beverage nor the plants used to prepare it are mentioned in the Convention on Psychotropic Substances of United Nations. Nonetheless, the use of ayahuasca compounds is controlled in some European countries, namely, France, where DMT, THH, HML, and HMN are illegal. In several other European countries, however, only DMT is illegal, according to a review by Horák et al. [29].

## 2. N,N-Dimethyltryptamine

DMT is a simple molecule of low molecular weight (188.27 g/mol) and hydrophobic character (logP = 2.573) [26]. It is similar to naturally occurring molecules in the body, such as serotonin and melatonin, rapidly crossing the blood–brain barrier [30]. This compound has high affinity for some neuroreceptors, binding to them and triggering very robust responses [26]. DMT is the main psychoactive component of ayahuasca and can be present in a large number of plants [26,31,32]. As previously stated, DMT is more common in the leaves of *P. viridis*, where the concentration of DMT varies between 0.1% and 0.66% of the leave dry weight, depending also on the plant and the time of the day in which they are collected [33,34]. Generally, each preparation of ayahuasca can contain between 8.8 mg and 42 mg of DMT, causing hallucinogenic effects with doses higher than 0.2 mg/kg [26,33]. Intraperitoneal LD_50_ in rats is reported as 47 mg/kg. In the case of intravenous administration, the LD_50_ in rats is 32 mg/kg, while in humans, it is estimated to be approximately 1.6 mg/Kg [33]. With regard to DMT when ingested through ayahuasca preparations, the LD_50_ estimated is 8 mg/Kg, since not all the amount consumed will be bioavailable [33].

### 2.1. DMT Pharmacokinetics

The effects of DMT vary greatly depending on how it is administered. Smoking is the preferred route for its recreational consumption, although the intravenous route is widely used [26,35]. When consumed by the latter modality, the psychoactive effects of this substance are rapid, reaching a maximum intensity 5 min after injection and decreasing in the next 30 min [26]. DMT is rapidly metabolized by the enzyme monoamine-oxidase A (MAO-A) present in the liver, the half-life of this substance being approximately 5 to 15 min [36,37]. Due to this rapid metabolization, only about 1.8% of the dose of DMT injected into the bloodstream can be measured. Likewise, only 0.16% of the injected dose is detected in urine [26]. When this substance is smoked or inhaled, identical psychoactive effects manifest [26,33,38]. They begin to manifest immediately, peaking in just a few minutes and disappearing about 30 min later. However, data on the effects of DMT consumed in the smoked form remains scarce [35]. When taken orally, DMT is rapidly degraded by MAO-A present in the intestine and liver, which prevents its access to the bloodstream [38,39,40,41]. As previously mentioned, whenever DMT is ingested along with MAO-A inhibitors, such as in the case of ayahuasca tea admixtures, it is able to access the bloodstream and rapidly reach the brain, thus exerting its psychoactive effects [26,33]. The main metabolites identified in this mixture are indoleacetic acid, 2-methyl-1,2,3,4-tetrahydro-β-carboline, DMT-N oxide, N-methyltriptamine, 1,2,3,4-hydro-β-carboline, and tryptamine [42,43,44,45,46,47].

### 2.2. DMT Pharmacodynamics

DMT has high affinity for receptors that are part of the serotonergic system. Many of its effects are due to interactions with such receptors [26]. Serotonin 1A (5-HT 1A) receptors are coupled to Gi proteins that mediate inhibitory neurotransmission and are usually expressed in serotonergic neurons as well as in specific cells of the cortical and subcortical regions [48,49]. DMT binds with some affinity to this type of receptors, acting as an agonist [50]. Agonists of these receptors, such as DMT, have been demonstrated to have antidepressant and anxiolytic activity [51,52]. Possibly, these effects are a result of the desensitization of these receptors during chronic consumption of these agonist substances [26]. In contrast, Serotonin 2A (5-HT2A) receptors are the best-characterized receptors to date; they are coupled to the Gq protein and mediate excitatory neurotransmission [26]. DMT leads to the stimulation of this receptor, acting as an agonist [26,44]. According to the literature, the hallucinogenic effects of DMT are due to this agonist effect on 5-HT2A18 receptors [53]. Studies conducted by Aghajanian and collaborators [54,55] showed that DMT is responsible for the stimulation of 5-HT2A receptors, leading to an increased response produced by spontaneous excitatory post-synaptic currents in cortical pyramidal neurons.

In another study, Mckenna and coworkers [56] found that DMT has small methyl groups that are responsible for a high affinity towards 5-HT2A receptors. Over time, this group of receptors maintains its sensitivity to DMT, which may justify the fact that the human body does not develop tolerance to this substance [26]. Another type of serotonergic receptor coupled to Gq proteins is 5-HT2C. DMT has only low affinity for these receptors, acting as a partial agonist [26]. In addition, over time, 5-HT2C receptors lose sensitivity to DMT [53]. The affinity of DMT for other serotonergic receptors, such as 5-HT6, 5-HT7, and 5-HT1D, is also described [50,57,58]. However, further studies are required to understand the possible effects of this affinity. A study by Bunzow and coworkers [59] suggested that DMT also interacts with the trace amine-associated receptor 1 (TAAR1). In this study, they resorted to the HEK293 cell line expressing TAAR1 and demonstrated that DMT activates TAAR1 by increasing cAMP production [59].

DMT has also affinity for the sigma-1 receptor. This affinity is about 100 times less than that for the above-mentioned 5-HT 2A receptors; nevertheless, this substance is one of the few known endogenous agonists of such receptor [60]. Szabo and coworkers [61] have recently shown that DMT triggers a mechanism through the sigma-1 receptor that protects cortical neurons from the effects of oxidative stress. Sigma-1 receptors are also targeted in the treatment of anxiety and depression [62]. Given that DMT produces antidepressant responses, it is possible that this receptor is involved in the mechanism that triggers these effects [26].

The effects of DMT on the cholinergic and dopaminergic systems have also been investigated, but on a smaller scale than those on the serotonergic system. Therefore, the information available is scarce [26]. DMT has a reduced binding affinity for dopamine receptors, compared to other substances [63]. Back in the 1970s, a study by Smith [64] suggested that DMT leads to increased dopamine production. Yet, in a different study by Haubrich and coworkers [65], it was shown that the dopamine levels in rat brains decreased after DMT administration. In the same study, it was also possible to demonstrate that when DMT was administered to rats, acetylcholine levels decreased in the striatum, but no change was observed in the cortex [65]. In a more recent study, where 18 volunteers were administered between 0.6 and 0.85 mg DMT per kg body weight, it was possible to verify that DMT exerts an agonistic effect on dopamine receptors [66].

Also the serotonin transporter (SERT) and the vesicular monoamine transporter (VMAT) are affected by DMT. Sangiah and colleagues [67] examined rat brain slices and found DMT accumulation achieved through an active transport mechanism. In another study by Cozzi and coworkers [68], it was possible to conclude that DMT acts as a substrate for both transporters.

### 2.3. Adverse Effects of DMT

When DMT is consumed, some physical effects such as nausea, vomiting, and diarrhea are common [26,33]. Also, increased heart rate and blood pressure were observed [27]. A study by Riba and coworkers [69] showed that an oral dose of 1 mg/kg DMT is sufficient for the occurrence of these effects. Otherwise, if DMT is administered intravenously, only doses between 0.1 and 0.2 mg/kg are required to produce these effects. Another study showed that 2 min after intravenous DMT administration, systolic blood pressure increased by about 35 mmHg and diastolic blood pressure increased by about 30 mmHg [27]. In the same study, there was also an increase in heart rate by about 26 beats per minute [27].

Other symptoms commonly manifested when consuming DMT are visual hallucinations and delirium [26,33]. DMT can cause emotional distress and may lead to psychosis or even schizophrenia [26,33]. Studies have shown that in patients with schizophrenia, the amount of DMT in urine and blood is above normal [70,71]. Years later, Ciprian-Ollivier and coworkers [72] hypothesized that DMT would lead to a gradual degradation of the cognitive processes. However, these findings are somewhat controversial, since another study concluded that increased levels of DMT have a calming effect and suppress psychotic activity [73]. Also, a sensation of relaxation caused by DMT has been described in the literature. Gillin and coworkers [74] carried out a clinical trial with intramuscular administration of DMT to healthy patients and reported that a great part of the participants experienced a feeling of relaxation. The same results were obtained in another study by Strassman and Qualls [27] with intravenous administration of DMT to healthy patients. Other studies indicate that the action of DMT on serotonergic neurons results in depression and anxiety [21,75,76,77]. However, there is, once again, some controversy in these results, since other studies suggest that DMT has anti-depressant properties and enhances a positive mood [18,27,33,74].

Psychedelic substances are commonly associated with dependence, but this does not apply to DMT, considering that, to the present date, there are no reports in the literature that this substance shows withdrawal symptoms when it is not consumed [26,33].

## 3. β-Carbolines Alkaloids

B-CA derive from tryptophan amino acid, presenting a heterocyclic and dehydrogenated structure. They are synthetized through the condensation of indolamines along with aldehydes or α-keto acids [4,78]. According to Moura and coworkers [79], B-CA have a broad spectrum of action in human organs and can be found not only in animals, but also in many plants and fungi [80]. Besides, they can function as endogenous compounds in some mammalian species [4].

Harmaline was first isolated from the seeds and roots of *P. harmala L* (Zygophyllaceae), in which it appeared to be the major compound (5.6% *w/w* on the seeds). However, this plant also contains HMN, harmalol (HLOL), and THH, mainly in the seeds and roots [81], in quantities varying between 2% and 5% [82].

In 1999, Ott decided to evaluate the combined effects of DMT and HMN on his own body. Therefore, he started by taking 20 mg of DMT and 40 mg of HMN and then increased HMN doses, having discovered that 1.5 mg/kg of this compound exerted effects when combined with DMT, whereas DMT alone had little or no effects [83].

One of the mechanisms of action of B-CA is the reversible inhibition of MAO activity. These enzymes, present in the mitochondria membrane, are of two types: MAO-A and MAO-B. Their main functions are the oxidation of endogenous and exogenous amine substrates such as mono- and polyamines, and the modification of amino acids within proteins [84,85]. They can be found in the brain, kidney, spleen, and intestines [86]. In the brain, MAO-B is more abundant then MAO-A, but both are responsible for the oxidative deamination of several neurotransmitters, such as dopamine, serotonin, and norepinephrine [87,88], although these compounds have been reported to be specific substrates of MAO-A [52].

On the one hand, B-CA can undergo an N-methylation reaction on the second position, and the derived compound is an analogue of the 1-methyl-4-phenylpyridinium ion (MPP+), which can have mitochondrial cytotoxic effects, since its accumulation can lead to oxidative stress, which blocks the complex I of the mitochondrial electrons transport chain [89]. Diversely, some researchers suggested that B-CA has anti-oxidant properties against reactive oxygen species (ROS), possibly reducing cell damage [78].

When comparing the effects of harmine with those of other drugs (Lysergic Acid Diethylamide (LSD) and mescaline) in humans and animals, Naranjo found that harmine oral administration (20–50 mg) produced psychedelic effects beginning after 20–30 min and lasting up to 6–8 h, with the greatest effect after about 30 min to 1 h. The same did not occur when harmine was administered through intramuscular injection (10–20 mg) [90]. In this case, the effects started 5–10 min and lasted up to 3–5 h, reaching a maximum 30 min after administration. Compared to the effects of the other drugs, the effects of harmine were very much similar on a qualitative basis, but with different magnitude [90], so that harmine was described as the most “horrible” and “paranoid” drug, causing mydriasis, salivation, lacrimation, hyperthermia, hyperglycemia, hypotension, and other manifestations. All compounds tested caused aggressive behaviors in both humans and animals, as well as psychedelic and emotional effects [91].

Later studies found that harmaline psychedelics effects were achieved only when intravenous doses were higher than 1 mg/kg and were practically immediate after injection; in contrast, they required oral doses higher than 4 mg/kg, manifesting about one hour after harmaline consumption [91]. Some of the subjective effects reported by the author were nausea and vomiting, different types of vision, feeling of floating in air. Although this study was essential to uncover the potential effects of B-CAs, it is important to consider that the method it used was archaic compared to today’s technology and methodology. Despite all existing researches, more studies on B-CA need to be performed to understand in depth its action mechanism and its biochemical effects on the human body.

Since the interest in these compounds is increasing with the increase of ayahuasca consumption and because DMT is responsible for ayahuasca psychedelic effects, publications regarding the pharmacokinetics and dynamics of isolated B-CA remain scarce in the literature. Yet, it is already known that DMT and B-CA behave synergistically.

## 4. Methods of Quantification of Ayahuasca

As the consumption of ayahuasca tea and similar plants increases, new challenges and the development of new techniques in the forensic and toxicological fields become of utmost importance. One of the objectives of this review is a compilation of various existing and published procedures for ayahuasca quantification. This review reports the analysis on biological specimens such as urine and plasma, after consumption of ayahuasca teas or preparations.

The first work developed for the quantification of DMT and B-CA was performed by Yritia and coworkers. [92]. In this work, DMT present in plasma was extracted using liquid–liquid extraction (LLE) with n-pentane and was quantified using a gas chromatographer coupled to a nitrogen–phosphorus detector. Retentions of 74% and a quantification limit (LOQ) of 1.6 ng/mL were obtained. On the other hand, harmine, harmaline, and tetrahydroharmine, as well as harmine and harmaline O-demethylation metabolites, were quantified in the plasma using high-performance liquid chromatography (HPLC) coupled to a fluorescence detector, having previously performed a solid-phase extraction (SPE). Recoveries above 87% and an LOQ of 0.5 ng/mL for harmine, 0.3 ng/mL for harmaline, 0.3 ng/mL for harmol and harmalol, and 1.0 ng/mL for THH were obtained. In both processes, a good linearity was observed in the concentration ranges evaluated for DMT (2.5–50 ng/mL) and for β-carbolines (0.3–100 ng/mL). Later, in 2008, Pires and coworkers [93] developed a new method for the simultaneous quantification of DMT and B-CA. The alkaloids were extracted by SPE (C_18_) and quantified by gas chromatography with nitrogen–phosphorus detection. The method performance was linear in the concentration range of 0.02 to 4.0 mg/mL (*r^2^* > 0.99), with LOQ being 0.02 mg/mL. In 2012, Oliveira and coworkers [94] quantified the constituents of ayahuasca (DMT and B-CA) in human plasma. For this purpose, the analytes were extracted by SPE (C18) and quantified using liquid chromatography coupled to mass spectrometry (LC–MS/MS), determining LOQs lower than 0.5 ng/mL for all analytes. The following year, Gaujac and coworkers [3] combined a solid-phase microextraction technique (SPME) in headspace mode with gas chromatography coupled to ion trap mass spectrometry (GC–IT–MS) in order to quantify DMT. The method showed accuracy values between 71% and 109% and good linearity (1.56 to 300 mg / L, *r^2^* = 0.9975). The LOQ was 9.5 mg/L, and the limit of detection (LOD) was 0.78 mg/L. Finally, in 2014, Pichini and coworkers [95] developed a method for the detection of various substances, including DMT, in hair samples, using ultra-high-pressure liquid chromatography–tandem mass spectrometry. Initially, the hair was washed with methyl alcohol and diethyl ether, and internal standards were subsequently added. The samples were then treated with VMA-T M3 reagent (acidic aqueous buffer) for 1 h at 100 °C, and, after cooling, 100 μL of M3 extract was diluted with 400 μL of water, and 10 μL was injected into the apparatus. A reverse-phase column maintained at room temperature was used, and elution was performed in linear gradient with 0.3% formic acid in acetonitrile ammonium formate (5 mM pH 3). The method performance was linear from LOQ of 0.03–0.05 ng/mg up to 10 ng/mg of hair. Recovery was between 79.6% and 97.4%. Table 1 summarizes the methods published about the determination of ayahuasca components in several human biological matrices and plant materials.

## 5. In Vivo and In Vitro Studies of Ayahuasca Compounds

For many years, studies on ayahuasca or its chemical compounds have been focused on humans and have been mainly based on inquiries or clinical trials. Therefore, the results obtained are subjective, and little is known about the biochemical and physiological effects. Some of the symptoms reported after constant ayahuasca consumption are feelings of confidence and optimism and, on a psychiatric level, a reduction on the lack of concentration, sleep deprivation, irritability, and depression [25,105]. In a recent study, whose main goal was to understand the possible reproductive effects of the beverage in male Wistar rats [106], researchers used the same dose (1×) and doses 2, 4, and 8 times higher (2×, 4×, 8×) than the dose used in a ritual. The content of the 1× dose was 0.146 mg/mL of DMT, 0.12 mg/mL of harmaline, and 1.56 mg/mL of harmine. It was observed that only the animals exposed to the 8× the dose suffered from stress, showing vocalization during the gavage procedure, piloerection, tremors, and weight loss. A decrease of the will to eat was mostly observed in animals treated with the 4× or 8× dose. Indeed, two animals treated with the 8× dose died. They found that such results indicate that exposure to higher doses could be representative of chronic toxicity in rats. Moreover, for the 4× dose, it was observed a reproductive toxic effect, and for the 8× dose, a reduction of the testis size in male rats, without further morphological changes.

In vitro studies concerning the effects or therapeutics potential of ayahuasca and/or its major compounds are scarce. Moreover, to the best of our knowledge, no in vitro study has been done evaluating the effects of traditional ayahuasca (*B. caapi* + *P. viridis*) or other plants used as analogues, such as *P. harmala* or *M. tenuiflora*. In addition, the published works only focus on one of the compounds and do not report the overall ayahuasca effects. Still, we tried to make a synthetic review of what has been published so far.

In 2010, Samoylenko and coworkers [107] conducted a study with different mammalian cellular lines, including human cancer cell lines (SK-MEL, KB, BT-549, and HepG2), non-human primate kidney fibroblasts (VERO), and pig kidney’s epithelial cells (LLC-PK11). The cytotoxicity at a concentration of 100 µg/mL was tested toward different enzymes, namely, acetylcholinesterase (AChE), butylcholinesterase (BuChE), and catechol-O-methyl transferase (COMT). No negative effects on SK-MEL, BT-549, and monkey’s VERO cell line were observed [107]. These authors also studied MAO inhibition by HMN and HML, concluding that these compounds may have therapeutic potential in the treatment of Parkinson’s Disease [107].

In a different study using just *B. caapi* alkaloids, conducted by Morales-García and coworkers, it was found that HMN, THH, and HML can stimulate adult neurogenesis, which is the mechanism that develops new functional neurons from progenitor cells. This was grounded on the fact that these B-CA have an anti-depressant effect and are associated with the capacity of this drugs to stimulate neurogenesis. It was concluded that these chemical substances can promote proliferation, migration, and differentiation of progenitor cells from the sub-ventricular zone to the sub-granular zone, which are the main areas of the brain where neurogenesis happens [14].

## 6. Conclusions and Further Perspectives

Over the years, the consumption of ayahuasca all over the world has increased not only in shamanic and religious rituals, but also for recreational purposes. Therefore, many psychological studies have been conducted in order to evaluate ayahuasca effects on a mental and subjective level. Given that, clinical and in vivo studies have also increased, evaluating ayahuasca biological effects particularly in the CNS, one of its main targets.

Even though it is known that the components of the beverage, mainly B-CA and DMT, exert their effects by inhibiting MAO-A (B-CA) and through DMT actions after entering the bloodstream, little is known of the effects at the cellular level, and few in vitro studies have been conducted. The ones considered in this review describe positive effects on proliferation and development of new brain cells (neurogenesis). Nonetheless, one may wonder about the cytotoxicity of ayahuasca, not only because the beverage is consumed for long periods of time, as the rituals take place twice a month, but also because recreational use of this beverage is becoming more prominent in western populations. Thus, further investigation in this sense is needed. The main goal of this article was to review in vitro studies done with these compounds and to try to understand their effects on cells.

It is of outmost importance to have highly sensitive and selective analytical methods in order to identify these compounds in several biological specimens. This is crucial in the clinical field, mainly in the countries that consume ayahuasca regularly, since it can help differentiate ayahuasca compounds intoxication from intoxications due to other drugs of abuse. In the same way, it is important to continue developing studies both in vitro and in vivo to acquire further knowledge on the biological behavior of ayahuasca compounds.

Still, more studies need to be performed to establish whether ayahuasca effects are beneficial or if they can cause severe and irreversible damages after long ayahuasca exposure/intake.

## Figures and Tables

**Table 1:** Bioanalytical procedures for the determination and quantification of the major compounds of ayahuasca in biological samples and ayahuasca teas or preparations.

Analyte	Matrix	Sample Preparation	Detection Mode	Stationary and Mobile Phase	Recovery (%)	LOD; LOQ	Concentrations of the Compounds	Reference
DMT, HMN, HML, THH	Plasma	HPLC–NPD: Protein precipitation; GC–NPD: LLE (n-buthylchloride)	HPLC–FLD (HMN, HML, THH); GC–NPD (DMT)	HPLC-NPD: Supercosil LC-DB-8 (15.0 × 4.6 mm i.d., 5 mm); Mobile Phase: Methanol/acetonitrile/ ammonium acetate 0.1M pH = 6.9 (HPLC–FLD); GC–NPD: DB-1 and DB-17	n.a	0.5 ng/mL; 5 ng/mL (DMT); 0.1 ng/m; 2.00 ng/mL (HMN) 0.05; 1.00 ng/mL (HML) 0.1 ng/mL; 1.9 ng/mL (THH)	222.3 ng/mL (HMN); 9.4 ng/mL (HML) 134.5 ng/mL (THH)	[96]
DMT, HMN, HML, THH, harmol and HLOL	Plasma	LLE (n-pentane) (DMT); SPE (HMN, HML, THH and THH O-demethylation metabolites)	GC–NPD (DMT); HPLC–FLD (HMN, HML, THH and THH O-demethylation metabolites)	GC–NPD: 5% phenyl-methylsilicone (12 m × 30.2 mm × 0.33μm film thickness) (DMT) HPLC–FLD: Kromasil 100 C_18_ (5 μm, 150 × 34 mm); Mobile phase: Solvent A: mixture ammonium acetate buffer (50 mM, pH 8.0) (63:37 *v*/*v*) and acetonitrile/methanol (20:30 *v*/*v*) and Solvent B: mixture of acetonitrile/methanol (20:30 *v*/*v*) (HMN, HML and THH); Solvent A: mixture ammonium acetate buffer (50 mM, pH 6.3) (73:27 *v*/*v*) and acetonitrile/methanol (20:30 *v*/*v*); Solvent B: acetonitrile/methanol (20:30 *v*/*v*) (harmol and HLOL)	74 (DMT); >87 HMN, HML, THH and THH O-demethylation metabolites)	n.a; 1.6 ng/mL (DMT), 0.5 ng/mL (HMN), 0.3 ng/mL (HML), 0.3 ng/mL (harmol and harmala) and 1.0 ng/mL (THH)	0.53 mg/mL (DMT); 0.9 mg/mL (HMN); 0.06 mg/mL (HML); 0.72 mg/mL (THH)	[92]
DMT, THH, HML and HMN	Plasma	SPE (C_18_)	LC–MS/MS (ESI)	Phenomenex Synergi Hydro-RP80A (50 × 2.0 mm, 4 μm); Mobile phase: Solvent A: mixture of aqueous solution of ammonium formate (5 mmol/L) with formic acid (0.1%); Solvent B: methanol and formic acid (0.1%)	88.4–107.7	0.1 ng/mL; 0.2–0.4 ng/mL	1.2–19.8 ng/mL (DMT); 1.0–15.6 ng/mL (HMN); 2.7–15.7 ng/mL (HML) and 27.1–71.4 ng/mL (THH)	[94]
DMT	Hair	Hydrolysis (M3 reagent)	UHPLC–MS/MS (ESI)	Acquity UHPLC HSS C_18_ (2.1 mm × 150 mm, 1.8 µm); Mobile phase: solvent A: formic acid in acetonitrile (0.3%), Solvent B: ammonium formate (5 mM, pH 3)	79.6–97.4	0.01–0.02 ng/mg; 0.03–0.05 ng/mg	5.6 ng/mg	[95]
DMT, THH HML and HMN	Ayahuasca preparations	SPE (C_18_)	GC–NPD	HP Ultra-2 (25 m × 0.2 mm × 0.33 μm) and Solvent A: formic acid in acetonitrile (0.3%); solvent B: ammonium formate (5 mM, pH 3)	68.4–99	10000 ng/mL; 20000 ng/mL	0.31–0.73 mg/mL (DMT); 0.37–0.83 mg/mL (HMN); 0.64–1.72 mg/mL (HML) and 0.21–0.67 mg/mL (THH)	[93]
DMT	Ayahuasca beverages	SPME (polydimethylsiloxane/divinylbenzene fiber)	GC–IT-MS (EI)	Supelco SLB-5 MS (30 m × 0.25 mm, 0.25 mm film thickness)	71–109	780 ng/mL; 950 ng/mL	0.17–1.14 mg/mL	[3]
DMT, THH, HMN and HML	Ayahuasca beverages	Dilution with methanol/water (1:1) and direct injection	LC–MS/MS (ESI)	Acquity™ UPLC BEH C_18_ (50 mm × 2.1 mm, 1.7 μm); Mobile phase: water (90%); solvent B: methanol (10%)	n.a	n.a; 150 ng/mL (DMT); n.a; 350 ng/mL (THH); n.a; 600 ng/mL (HMN) and n.a;100 ng/mL (HML)	62–340 µg/mL (DMT); 402–3308 µg/mL (THH); 414–1816 µg/mL (HMN); 44–420 µg/mL (HML)	[97]
DMT; HML; HMN	Ayahuasca beverage	LLE (10 mL diethyl ether)	GC–MS (ion trap) (EI)	Chrompack CP–SIL 8CB-MS (30 m × 0.25 mm × 0.25 μm)	n.a	n.a	0.24 mg/mL (DMT); 0.06 mg/mL (HML); 0.34 mg/mL (HMN)	[98]
DMT; THH; HMN; HML; HLOL; harmol and metabolites	Ayahuasca preparations	Dilution with mobile phase and direct injection	LC–MS/MS (ESI)	Zorbax Eclipse Plus HT C18 (1.8 µm × 4.6 × 50 mm (i.d.)); Mobile phase: Solvent A: formic acid (0.1% in water); Solvent B: Formic acid (0.1% in acetonitrile)	n.a	6.4; 210 ng/mL (DMT); 0.5; 210 ng/mL (THH); 0.5; 100 ng/mL (HMN); 2.8; 220 ng/mL (HML); 34.3;510 ng/mL (HLOL)	0.13–3.19 mg/mL (DMT); 1.22–11.90 mg/mL (THH); 0.91–16.14 mg/mL (HMN); 0.2186–1.55 mg/mL (HML); 0.0026–0.0310 mg/mL (HLOL); 0.0009–0.0633 mg/mL (harmol); 0.0052–0.0313 (N-methyltryptmine)	[99]
DMT; THH; HMN; HML; HLOL; harmol and various metabolites	Urine	Enzymatic hydrolysis (B-glucuronidase/sulfatase) of urine, dilution with mobile phase and direct injection	LC–MS/MS (ESI)	Zorbax Eclipse Plus HT C18 (1.8 µm × 4.6 × 50 mm (i.d.)); Mobile phase: Solvent A: formic acid (0.1% in water); Solvent B: Formic acid (0.1% in acetonitrile)	n.a	0.12; 5.00 ng/mL (DMT); 0.21; 5.00 ng/mL (THH); 0.18; 5.00 ng/mL (HMN); 0.07; 5.00 ng/mL (HML); 0.18; 5.00 ng/mL (HLOL)	0–0.6 µg/mL (DMT); 0–6.3 µg/mL (THH); 0–0.21 µg/mL (HMN); 0–0.53 µg/mL (HML); 0–14.16 (HLOL); 0.04–126.18 µg/mL (harmol)	[100]
DMT; THH; HMN; HML; HLOL; harmol and various metabolites	blood	Protein precipitation 96-well plates, dilution with mobile phase and direct injection	LC–MS/MS (HESI)	Zorbax Eclipse Plus HT C18 (1.8 µm × 4.6 × 50 mm (i.d.)); Mobile phase: Solvent A: formic acid (0.1% in water); Solvent B: Formic acid (0.1% in acetonitrile)	60.28–76.31	0.45; 1 ng/mL (DMT) 0.36; 1 ng/mL (THH); 0.25; 1 ng/mL (HMN); 0.22; 1 ng/mL (HML); 0.38; 1 ng/mL (HLOL); 0.3; 1 ng/mL (harmol)	0–15.09 ng/mL (DMT); 0–55.44 ng/mL (THH); 0–5.18 ng/mL (HMN); 0–4.53 ng/mL (HML); 0–3.27 ng/mL (HLOL); 0–5.55 ng/mL (harmol)	[101]
DMT; THH; HMN; HML	Ayahuasca preparation	direct injection	DART–HRMS	n.a	n.a	n.a	n.a	[102]
DMT; THH; HMN; HML	Leaves of *Psychotria viridis*	n.a	CE–LIF–MS (ESI)	Silica column (7.5 µm ID; 95 cm)	n.a	n.a	n.a	[103]
DMT; THH; HMN; HML	Ayahuasca beverage	n.a	NMR	n.a	70	12,500;12,500 ng/mL	400 µg/mL	[104]
DMT; THH; HMN; HML; HLOL; harmol and metabolites	Urine	Dilution with mobile phase and direct injection	LC–MS/MS (ESI)	Zorbax Eclipse Plus HT C18 (1.8 µm × 4.6 × 50 mm (i.d.)); Mobile phase: Solvent A: formic acid (0.1% in water); Solvent B: Formic acid (0.1% in acetonitrile)	n.a	n.a; 5 ng/mL for all compounds		[40]

CE: capillary electrophoresis; DART–HRMS: direct analysis in real-time–high-resolution mass spectrometry DMT: N,N-dimethyltryptamine; EI: electron ionization; ESI: electrospray ionization; GC–IT-MS: Gas chromatography coupled to ion trap mass spectrometry; GC–NPD: Gas chromatography coupled to a Nitrogen–Phosphorous Detector; HESI: heated electrospray ionization; HLOL: Harmalol; HML: Harmaline; HMN: Harmine; HPLC–FLD: High-performance liquid chromatography coupled to fluorescence detection; LC–MS/MS: liquid chromatography coupled to tandem mass spectrometry; LIF: laser-induced fluorescence; LLE: liquid–liquid extraction; LOD: Limit of detection; LOQ: Limit of quantification; n.a: not available; NMR: Nuclear magnetic resonance of proton; SPE: solid-phase extraction; SPME: solid-phase microextraction; THH: tetrahydroharmine; UHPLC–MS/MS: ultra-high-pressure liquid chromatography tandem mass spectrometry; CE–LIF–MS: Capillary electrophoresis-laser induced fluorescence-electrospray ionization-mass spectrometry.

## Abbreviations

5-HT receptors	5-Hydroxytriptamine receptors
AChE	acetylcholinesterase
B-CA	beta-carbolines alkaloids
BT-549	human breast cancer cell line
BuChE	butylcholinesterase
cAMP	cyclic adenosine monophosphate
CE	capillary electrophoresis
COMT	catechol-O-methyl transferase
CNS	central nervous system
DART-HRMS	direct analysis in real-time–high-resolution mass spectrometry
DMT	N, N-dimethyltriptamine
EI	electron ionization
ESI	electrospray ionization
GC	gas chromatography
GC-IT-MS	gas chromatography coupled to ion trap mass spectrometry
GC-MS	gas chromatography coupled to mass spectrometry
GC-NPD	gas chromatography coupled to nitrogen phosphorous detector
HEK293	human embryonic kidney 293 cells
HepG2	human liver cancer cell line
HESI	heated electrospray ionization
HLOL	harmalol
HML	harmaline
HMN	harmine
HPLC	high-performance liquid chromatography
HPLC-FLD	high-performance liquid chromatography coupled to fluorescence detector
IS	internal standard
KB	human HeLa contaminant carcinoma cell line
LC-MS/MS	liquid chromatography coupled to tandem mass spectrometry
LD50	lethal dose 50
LIF	laser-induced fluorescence
LLC-PK11	pig kidney’s epithelial cells
LLE	liquid–liquid extraction
LOD	limit of detection
LOQ	limit of quantification
LSD	lysergic acid diethylamide
MAO	monoamine oxidase
n.a.	not available
NMR	nuclear magnetic resonance of proton
MS	mass spectrometry
R2/r2	determination coefficient
SERT	serotonin transporter
SK-MEL	human melanoma cell Line
SPE	solid-phase extraction
SPME	solid-phase microextraction
TAAR1	trace amine-associated receptor 1
THH	tetrahydroharmine
UDV	União do Vegetal
UHPLC-MS/MS	ultra-high-pressure liquid chromatography–tandem mass spectrometry
VERO	monkey kidney cell line
VMAT	vesicular monoamine transporter
